# Characterization, Stability and Antioxidant Activity of Vanilla Nano-Emulsion and Its Complex Essential Oil

**DOI:** 10.3390/foods13050801

**Published:** 2024-03-05

**Authors:** Fei Xu, Yucong Shi, Bin Li, Chengmei Liu, Yanjun Zhang, Junzhen Zhong

**Affiliations:** 1 Spice and Beverage Research Institute, Chinese Academy of Tropical Agricultural Sciences, Wanning 571533, China; 2 Key Laboratory of Processing Suitability and Quality Control of the Special Tropical Crops of Hainan Province, Wanning 571533, China; 3 State Key Laboratory of Food Science and Resources, Nanchang University, No 235, Nanjing East Road, Nanchang 330047, China

**Keywords:** vanilla, compound essential oil, nano-emulsions, flocs

## Abstract

As a natural flavoring agent, vanilla essential oil has a special aroma and flavor, but its volatility and instability limit its value. Therefore, in this study, vanilla essential oil was compounded with cinnamon essential oil to prepare nano-emulsions (composite nano-emulsions called C/VT and C/VM), and the stability of the composite essential oil emulsions was investigated. Transmission electron microscopy (TEM) images showed that the nano-emulsions were spherical in shape and some flocs were observed in C/VM and C/VT. The results showed that the average droplet sizes of C/VM and C/VT increased only by 14.99% and 15.01% after heating at 100 °C for 20 min, and the average droplet sizes were less than 120 nm after 24 days of storage at 25 °C. Possibly due to the presence of reticulated flocs, which have a hindering effect on the movement of individual droplets, the instability indices of C/VM and C/VT were reduced by 34.9% and 39.08%, respectively, in comparison to the instability indices of C/VM and C/VT. In addition, the results of antioxidant experimental studies showed that the presence of composite essential oil flocs had no significant effect on the antioxidant capacity. These results indicate that the improved stability of the composite essential oil nano-emulsions is conducive to broadening the application of vanilla essential oil emulsions.

## 1. Introduction

Vanilla (*Vanilla planifolia* Andrews) is a typical tropical spice plant. Vanilla essential oil (EO) has a unique aroma, strong fragrance and other characteristics, can be used for daily use in products to add a unique aroma, but also can be used as a natural food additive, for flavoring and aromatizing food and beverages [1]. In addition, it also has a variety of health benefits such as antioxidant, antimutagenic and hypolipidemic activity [2]. The demand for products containing natural plant essential oil additives has increased dramatically due to dietary and health concerns [3]. However, the use of essential oils in foods has been limited by drawbacks such as high volatility, poor water solubility and issues with storage. Currently, most of the research on vanilla EO focuses on the study of the volatile compounds that contribute to the understanding of the complex aroma of vanilla, but there are fewer reports on the use of natural vanilla EO as an additive in products, which becomes a promising challenge [4,5].

Emulsions are used as bioactive component delivery systems to improve the stability of active components and overcome problems such as bioavailability. Emulsions can include, e.g., microcapsules, nano-emulsions, Pickering emulsions and liposomes. Recently, Wang et al. [6] prepared a Pickering emulsion of 5% vanilla essential oil phase and 2.5% Osa-starch using ultrasonication, and the minimum droplet size of the droplets measured 0.456 μm. Qiao et al. [7] used ultrasonic cell crushing and Tween-80 as an emulsifier to produce stable nano-emulsions of *Zanthoxylum bungeanum* Maxim essential oil with an average droplet size of less than 150 nm, improving its bioavailability. Compared to Pickering emulsions, nano-emulsions are thermodynamically unstable and kinetically stable systems with small droplet sizes (<200 nm), a homogeneous dispersion (0.1–0.4) and a high surface free energy, which acts to reduce van der Waals interactions to reduce flocculation and coalescence [8,9]. In addition, the stability of sodium caseinate-EGCG composite stabilized fish oil emulsions was increased by high-pressure microfluidization treatment, as reported by Tang et al. [10] Therefore, microfluidization was chosen to prepare stable nano-emulsions in this experiment to provide a new method to improve the stability of vanilla EO and expand its application.

According to Jiménez et al. [11], cinnamon essential oil nano-emulsions have higher physical stability and antioxidant and antimicrobial properties compared to pure pepper essential oil. Stable clove/cinnamon nano-emulsions with good antimicrobial properties were prepared using the emulsion phase inversion (EPI) method, as reported by Zhang et al. [12]. However, there are fewer literature reports on the exploration of the stability of compounded essential oil nano-emulsions. In addition, the differences between the physicochemical and antioxidant properties of vanilla essential oils from three different origins have been reported [13], whereas the differences in the emulsifying properties of vanilla essential oils from different origins have not been reported. Therefore, the novel aim of this experiment is to produce nano-emulsions with two vanilla essential oils of different origins and then compound them with cinnamon essential oil to improve the stability of the vanilla nano-emulsions and explore their stability under different conditions.

In this experiment, a vanilla essential oil nano-emulsion system was prepared using a microfluidizer processor with Tween-80 and sodium caseinate (NaCas) as an aqueous phase. Moreover, the vanilla essential oil was compounded with a cinnamon essential oil at a ratio of 1:1 (*v*/*v*) to investigate the stability of the nano-emulsion under different environmental conditions. Firstly, the droplet size, PDI, zeta potential, turbidity and micro-morphology of the nano-emulsions were investigated using a nano-particle size meter, UV spectrophotometer and transmission electron microscope, then, the thermal, pH and centrifugal stability of the nano-emulsions were explored, and lastly, the scavenging rate of the nano-emulsions against DPPH and ABTS free radicals was investigated. This study is aimed at providing an attractive method for improving the stability of vanilla essential oil emulsions and broadening the range of applications of vanilla essential oil emulsions.

## 2. Materials and Methods

### 2.1. Materials

The vanilla essential oil was obtained through a donation from the Hainan Tropical Research Institute. The other materials were cinnamon oil (85%), medium chain triglycerides (MCT) and 2,2-diphenyl-1-plcrylhydrazyl (DPPH) (analytical pure, Shanghai yuan ye Bio-technology Co., Ltd., Shanghai, China). As well as casein sodium salt (NaCas), Tween-80, 2,2′-azino-bis (3-ethylbenzothiazoline-6-sulfonic acid) (ABTS) and K_2_S_2_O_8_ (analytical pure, Shanghai McLean Biochemical Technology Co., Ltd., Shanghai, China)

### 2.2. Preparation of Nano-Emulsion

The method of preparing nano-emulsions was slightly modified according to the relevant literature [14]. The aqueous phase, consisting of 95 mL of NaCas solution (1% *w*/*w*) and 0.8 mL of Tween 80, and the oil phase, consisting of 2.2 mL of MCT with 3 mL of EO, were used, and the formulation of the nano-emulsions was kept unchanged except for the type and proportion of essential oils. The aqueous and oil phases were stirred in a high-speed shearing machine (IKA T25 basic, Staufen, Germany) at 10,000 rpm for 5 min to obtain the crude emulsion. This was then homogenized five times at 10,000 psi using a microfluidizer (M-110EH30, Newton, MA, USA). After preparation, the nano-emulsions were stored at 4 °C before analysis.

C, VM and VT denote the nano-emulsions made from cinnamon essential oil, Madagascar vanilla orchid essential oil, and Tonga vanilla essential oil, respectively, and C/VM and C/VT denote the nano-emulsions made from cinnamon essential oil blended with Madagascar vanilla essential oil and Tonga vanilla essential oil in a ratio of 1:1 (*v*/*v*), respectively.

### 2.3. Measurement of Droplet Size, Polydispersity Index and Zeta Potential

A Zetasizer (Zeta-sizer NanoZS, Malvern Instruments, Malvern, UK) was used to determine the average droplet size, PDI and zeta potential of the nano-emulsions. The emulsions were diluted 100 times with deionized water to reduce the multiple scattering effect, and tested at 25 °C with an equilibrium of 120 s.

### 2.4. TEM

The microscopic morphology of the formed nano-emulsions was observed by transmission electron microscopy (JEOL 2100F, Tokyo, Japan). The nano-emulsions were placed on a copper grid and stained negatively with 10 μL drops of 3% phosphotungstic acid, then, the shapes of the nano-emulsions were measured under a 200 kV transmission electron microscope.

### 2.5. Turbidity Measurement

The turbidity of the nano-emulsion was determined by an ultraviolet–visible spectrophotometer (UV-1600, Mapada, Shanghai, China) at 600 nm and 25 °C. The prepared emulsion was diluted 10 times before measurement. The turbidity of the nano-emulsion was calculated as follows:

T = 2.303AD/L

where T = the turbidity (cm^−1^) of nano-emulsions, A = the absorbance value of diluent at 600 nm, D = the dilution factor (10) and L = the path length of the cuvette (cm).

### 2.6. Stability Analysis

#### 2.6.1. Storage Stability

A 5 mL sample of fresh emulsion was sealed at 25 °C and stored for 2, 4, 6, 12 and 24 days, respectively. The droplet size, PDI and zeta potential were measured at each storage time [15].

#### 2.6.2. Thermal Stability

The thermal stability of the nano-emulsions was determined according to Lili et al. [16], with slight modifications. The fresh emulsions were treated at different temperatures (25, 65, 85 and 100 °C) for 20 min, then cooled to room temperature (25 ± 2 °C), diluted 100 times with distilled water, and the corresponding droplet size, PDI and zeta potential were determined.

#### 2.6.3. pH Stability

A sample of 5 mL of the freshly prepared emulsion was adjusted to the desired pH (4~8) with 0.1 mol/L NaOH and HCl, then diluted 100 times with distilled water, and the droplet size and zeta potential were determined [15].

### 2.7. LUMisizer Analysis

The stability of the nano-emulsions was assessed using the LUMiSizer (LUMiFuge-111, Hannover, Germany). As the test time extended, transmission curves of the sample tubes were recorded. The SEPView 6.4 software allows for the analysis of instability indices and emulsion kinetics. The operating parameters used for the experimental application were as follows: 1 mL nano-emulsion, the rotation speed was 4000 rpm, light factor was 1, time interval was 10 s and the temperature was 25 °C [17].

### 2.8. Antioxidant Activity Assay

The experiments to assess DPPH radical scavenging were based on the method described by Chunwei et al. [18], with some modifications. A DPPH anhydrous ethanol solution (0.2 mM) was prepared, set aside and diluted with anhydrous ethanol to give an absorbance between 0.7 and 0.8 at 517 nm to obtain a DPPH working solution. A total of 100 µL of the DPPH working solution and 100 µL of the different concentrations (4–20 mg/mL) of sample solution were added to a 96-well plate and shaken well. The reaction was carried out at room temperature in the dark for 30 min and the absorbance was measured at 517 nm using a microplate reader (BioTek Synergy H1, Winooski, VT, USA). The DPPH radical scavenging rate (CR) was calculated using the formula:

$$\mathrm{DPPH}\;\mathrm{CR}\left(\%\right)=\left[1-\frac{A_{1}-A_{2}}{A_{0}}\right]\times 100\%$$

where *A*_0_ is the absorbance value of the control group without sample, *A*_1_ is the absorbance value of the reaction group and *A*_2_ is the absorbance value of the blank group without the DPPH.

The experiment to assess ABTS radical scavenging was based on the methods described by Chunwei, Fan, Wenxuan, Wupeng, Xiuzhu and ShuangKui [18], with some modifications. The prepared stock solutions of ABTS (7.4 mM) and K_2_S_2_O_8_ (2.6 mM) were mixed in a 1:1 ratio, reacted in the dark for 12 h and diluted so that the absorbance at 734 nm was between 0.7 and 0.8 to give an ABTS working solution. A total of 100 µL of the ABTS working liquid and 100 µL of the sample liquids of different concentrations (4–20 mg/mL) were added to a 96-well plate and shaken well. The reaction was carried out at room temperature and in the dark for 10 min, and the absorbance was measured at 734 nm using a microplate reader (BioTek Synergy H1, USA). The ABTS radical scavenging rate (CR) was calculated using the formula:

$$\mathrm{ABTS}\;\mathrm{CR}\left(\%\right)=\left[1-\frac{A_{1}-A_{2}}{A_{0}}\right]\times 100\%$$

where *A*_0_ is the absorbance value of the control group without sample, *A*_1_ is the absorbance value of the reaction group and *A*_2_ is the absorbance value of the blank group without the ABTS.

### 2.9. Statistical Analysis

This experiment used Origin 2022 and ImageJ software for image drawing and processing. Statistical analysis was performed according to the analysis of variance (ANOVA) and the least significant difference test (*p* < 0.05) using SPSS version 20. To ensure the accuracy of the experiment, each experimental group was carried out in parallel with three groups, and all data were expressed as mean ± standard deviation.

## 3. Results and Discussion

### 3.1. Droplet Size and PDI

**Figure 1:**
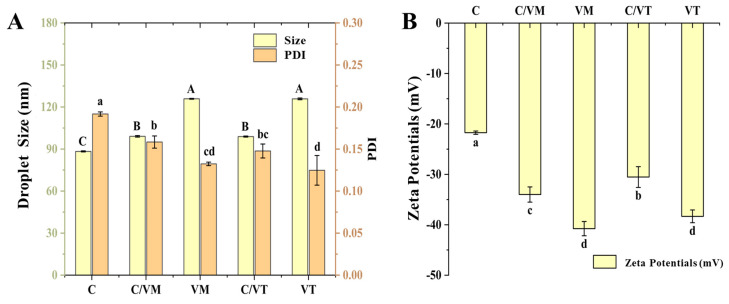
Droplet size, PDI values (**A**) and zeta potential (**B**) of nano-emulsions. a–d indicate significant differences (*p* < 0.05) in droplet sizes and potentials of different groups, and A–C indicate significant differences (*p* < 0.05) in PDI of different groups.

Droplet size is an important index for evaluating the stability of emulsions, and smaller droplet size appears to be one of the most important factors in stabilizing emulsions [19]. Therefore, the droplet size of nano-emulsions was investigated as shown in Figure 1. The size of the prepared nano-emulsions was less than 150 nm and showed a single peak droplet size distribution. The droplet size of C was 88.31 nm, and the droplet sizes of VM and VT were 125.80 nm and 125.86 nm. The droplet sizes of the composite nano-emulsions C/VM and C/VT were 99.07 nm and 98.89 nm, respectively. This indicated that the type of essential oil had a significant effect on the droplet size of the droplets (*p* < 0.05) [20], This may be due to the change in interfacial tension between the different types of oil and the aqueous solution; in the pre-experiment, cinnamon essential oil was tested to have an interfacial tension of 20 mN/m, whereas vanilla essential oils (Madagascar, Tonga) had interfacial tensions of 24 mN/m and 29 mN/m, respectively. Mixing the two essential oils changes their water/oil interfacial tension, resulting in the formation of much smaller droplet-size nano-emulsions.

Polydispersity, defined as the ratio of the standard deviation to the mean droplet size, responds to the dispersion of the droplets in the liquid, which implies that the droplets in nano-emulsions with low PDI values are uniformly distributed [21]. The experimental results show that the PDI value of C was 0.191, the values of VM and VT were 0.13 and 0.12, and the PDI values of the composite nano-emulsions C/VM and C/VT were 0.15 and 0.14, respectively, which are all less than 0.2, indicating that the droplets were uniformly dispersed [22].

### 3.2. Zeta Potential

The overall physicochemical, organoleptic and nutritional properties of many food emulsions are determined by the magnitude and sign of the electrical charge on the droplets. The zeta potential, which provides a high energy barrier and good electrostatic repulsion between droplets, is the effective surface potential of the droplet suspended in the medium [23]; therefore, the absolute value of the zeta potential is one of the factors influencing the stability of an emulsion. The zeta potentials of the nano-emulsions are shown in Figure 1B, the zeta potential of C was −21.73 mV, the zeta potentials of VM and VT were −40.76 mV and −38.33 mV, and the zeta potentials of C/VM and C/VT were −34 mV and −30.53 mV, respectively. These differences may be due to the addition of the cinnamon essential oil, the different states of the adsorption layer on the surface of the oil droplets of cinnamon and vanilla essential oils in response to NaCas [24], as well as differences in the free fatty acids in cinnamon and vanilla essential oils.

In summary, the selection of appropriate essential oils for compounding can lead to more stable nano-emulsions. For example, the compounded essential oil nano-emulsions showed a significant reduction in droplet size compared to the vanilla essential oil emulsions, and a marked increase in the absolute value of the zeta potential compared to the cinnamon essential oil.

### 3.3. TEM

**Figure 2:**
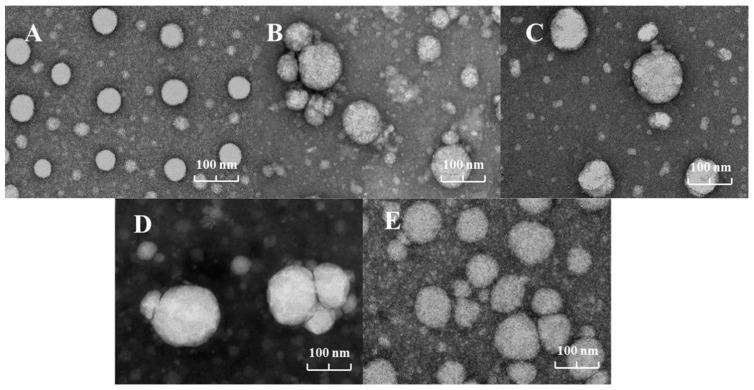
TEM morphology of the nano-emulsions prepared from each essential oil, (**A**–**E**) indicate C, C/VM, VM, C/VT and VT, respectively.

The micromorphological observations, which show the droplet sizes of the emulsions under intuitive conditions, showed that the nano-emulsions were brightly spherical and uniformly distributed in the dark environment, which is in agreement with the observation of Bagher and Dornoush [25]. The microscopic morphology of the specific droplet sizes of the nano-emulsions observed under intuitive conditions corresponds to the measured average droplet sizes. The oil phases of C/VM and C/VT were mixed with equal volume ratios of two different essential oils, as shown in Figure 2B,D, where we observed the formation of flocs in the nano-emulsions. A floc is a combination of two or more droplets that maintains the integrity of the droplets, which is fundamentally different from the process of coalescence [26].

### 3.4. Turbidity

**Figure 3:**
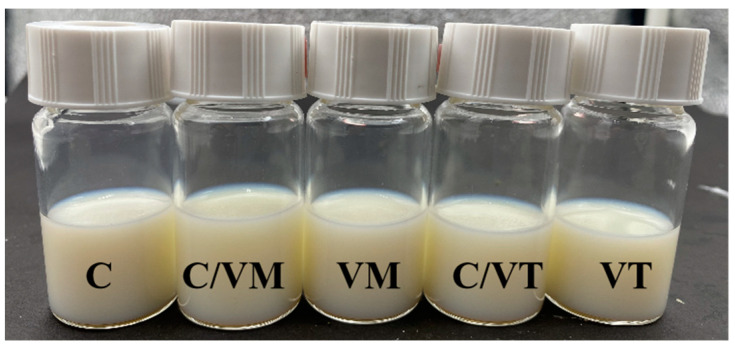
Freshly prepared nano-emulsion.

The color of an emulsion mainly depends on the scattering and absorption efficiency of the emulsion, where scattering is affected by the droplet properties (droplet characteristics, concentration), which determine the turbidity, opacity or brightness of the emulsion, and the absorption efficiency is affected by the coloring agent (absorption spectrum and concentration). As shown in Figure 3, the visual appearance of the newly prepared nano-emulsion corresponds to a milky white color, which may be attributed to the 1% (*w*/*w*) sodium caseinate as the continuous phase. The turbidity of the nano-emulsions was determined using a UV spectrophotometer as shown in Table 1, and the turbidity values of C, C/VM and VM were classified as 9.31, 11.73 and 23.22, which indicates that the turbidity of diluted emulsions as a function of wavelength can be used to determine the droplet size [27].

### 3.5. Stability Analysis

#### 3.5.1. Thermal Stability

**Figure 4:**
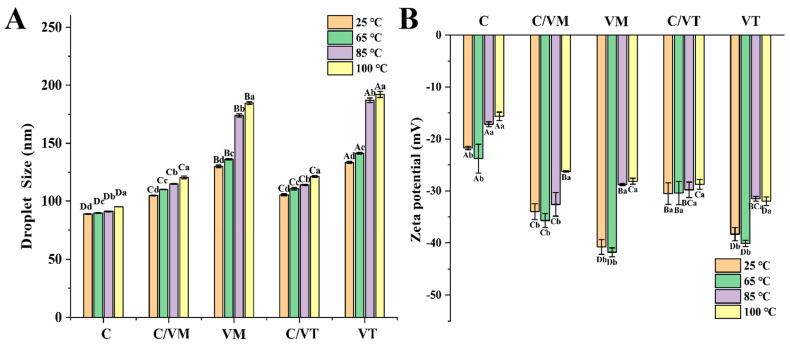
Droplet size (**A**) and zeta potential (**B**) of nano-emulsions. Lowercase letters indicate significant differences (*p* < 0.05) for different temperatures of the same nano-emulsion and capital letters indicate significant differences (*p* < 0.05) for different groups of nano-emulsions.

Nano-emulsions are thermodynamically unstable and increased temperatures can cause droplets to absorb energy in the nano-emulsion, resulting in the demulsification, coalescence and flocculation of the nano-emulsion [15]. As shown in Figure 4, the droplet size of C increased slightly (88.31 to 90.03 nm) at the test temperature, and the growth of the droplet size was very smooth. The droplet sizes were 173.96 nm and 184.53 nm for VM, and 187.03 nm and 192 nm for VT at 85 °C and 100 °C. The droplet sizes increased rapidly at high temperatures, which was consistent with the results of Hanen, Ben, A., Hiroko, Mitsutoshi and Riadh [20]. The reason for this phenomenon may be due to the increase in temperature which makes the large droplets move faster and the collision frequency and collision efficiency of the droplets increase, leading to the rapid coalescence of the droplets. For the compounded nano-emulsions, C/VM and C/VT showed acceptable increases in mean droplet size at all tested temperatures (104.7–120.4 nm and 105.4–121.23 nm). This might be due to the fact that the mixture of the two essential oils acted as a complementary effect, and, since the cinnamon essential oil emulsion had excellent thermal stability, the vanilla essential oil was compounded with the cinnamon essential oil to achieve the effect of improving the thermal stability of the emulsions (with respect to VM and VT).

#### 3.5.2. Storage Stability

Nano-emulsions have a long kinetic stability that can be maintained for months because of their small size and low dispersion index. When the nano-emulsion droplet size is less than 200 nm, the nano-emulsion is highly resistant to gravitational separation, which is ideal for the development of products with a long shelf life [28]. In this study, the nano-emulsions were stored at 25 °C for 24 d. The storage stability of each nano-emulsion was investigated using droplet size and zeta potential as indicators. Among them, the average droplet sizes of VM and VT (130.06 nm and 128.13 nm), as well as C/VM and C/VT (100.20 nm and 100.60 nm), remained in the nanoscale after 24 d of storage without phase separation, but the droplet size of C increased significantly after 24 d (1431 nm) and phase separation and oiling occurred. The formulations of the nano-emulsions were identical except for the different types of essential oils, so the possible origin of the differences in the stability of the nano-emulsions is Ostwald ripening [29]. This is in line with Wan, Zhong, Schwarz, Chen and Rao’s [29] experimental results, in which, of the five essential oil nano-emulsions, the cinnamon essential oil nano-emulsion showed a significant increase in droplet size after storage at 25 °C for 30 d. The thyme, peppermint and lemongrass nano-emulsions were relatively stable throughout the test period, and it is possible that these essential oils are relatively poorly soluble in water, which inhibited the growth of droplets through a compositional ripening effect. In addition, lemon oil with different chemical compositions provided the same experimental results [30]. Therefore, in the essential oil-blend nano-emulsions created in this experiment, it is possible that the addition of vanilla essential oil reduced the overall water solubility of the blend, leading to the inhibition of Ostwald ripening.

**Figure 5:**
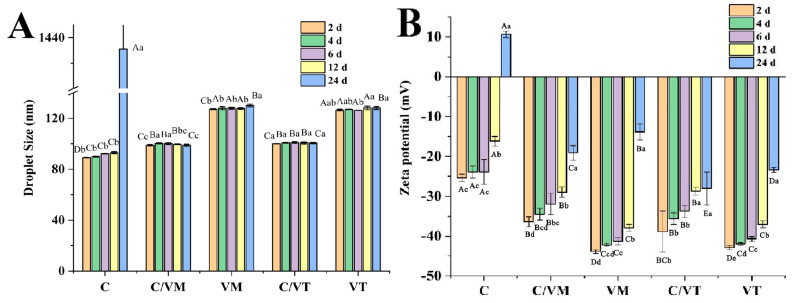
Droplet size (**A**) and zeta potential (**B**) of emulsions stored at 25 °C. Capital letters indicate significant differences between groups (*p* < 0.05) and lowercase letters indicate significant differences between storage times (*p* < 0.05).

As shown in Figure 5B, the absolute values of the zeta potentials of all the nano-emulsions decreased with the increase in storage time, indicating that the electrostatic repulsion between droplets tended to weaken with the increase in storage time. The zeta potential of C changed from a negative value to a positive value of 10.7 mV, and the associated droplet size increased to 1431 nm, indicating that after 24 days of storage at 25 °C the droplets of C had broken the emulsion and the system was destabilized. It is possible that droplet coalescence occurs during storage, where the membranes of neighboring droplets fuse, resulting in a variation of potential as well as a larger droplet size, which, given the difference in densities, ultimately leads to frosting under the action of gravity [31].

#### 3.5.3. pH Stability

**Figure 6:**
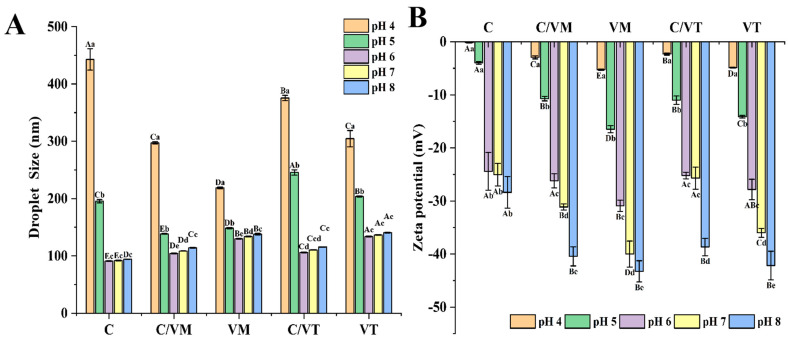
Droplet size (**A**) and zeta potential (**B**) of nano-emulsions at different pH values. Capital letters indicate significant differences between groups (*p* < 0.05) and lowercase letters indicate significant differences between storage times (*p* < 0.05).

The charge carried by the droplets has an important effect on the pH stability of emulsions where proteins are involved in emulsification, so the effect of different pH values on the droplet size and zeta potential of nano-emulsions was determined. As shown in Figure 6, the droplet size of the nano-emulsions did not show significant (*p* > 0.05) changes at pH 6–8. A remarkable increase (*p* < 0.05) in the droplet size of all the nano-emulsions could be observed when the pH was 4. The size of the droplets of C was 835.35 nm, the droplet sizes of VM and VT were 243.75 nm and 479.9 nm, respectively, and the droplet sizes of C/VM and C/VT were 693.85 nm and 523.15 nm, which was in agreement with the findings of Shi, Zhang, Chen and Wang [15]. As the pH value of 4 is close to the NaCas isoelectric point, the electrostatic charge on adsorbed proteins decreases, resulting in a low surface charge density and the loss of electrostatic stabilization [32], which may be the reason for the increase in droplet size. On one hand, this is because when the pH is close to the isoelectric point of NaCas (pH = 4.6) the electrostatic charge of NaCas is zero, which leads to a decrease in the solubility of the protein, restricting its ability to migrate to the water–oil interface and reducing the emulsifying capacity. Another aspect of the reason is that, at pH = 4, the number of hydrogen ions in the emulsion increases, the absolute value of the zeta potential decreases and the electrostatic repulsive force between droplets is not enough to overcome the attractive interactions between droplets, causing the droplet size to increase. As the pH value continues to rise, the absolute value of the zeta potential increases significantly, providing the droplets with a strong enough electrostatic repulsion to overcome the attractive interactions between droplets [33].

### 3.6. LUM Analysis

**Figure 7:**
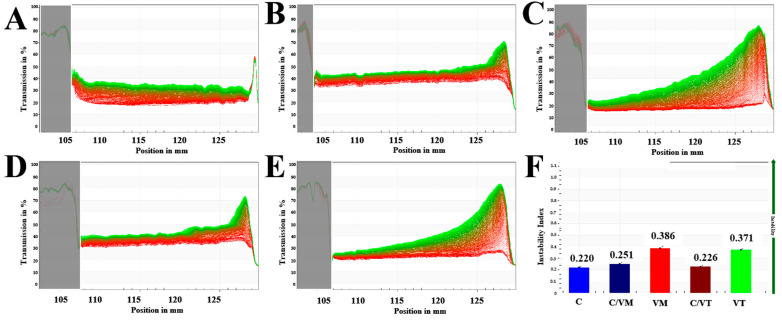
Transmission profiles of the nano-emulsions, (**A**–**E**) represent C, C/VM, VM, C/VT and VT, respectively; (**F**) represents the instability index of the nano-emulsions.

The evolution of the transmission curves of the nano-emulsions is shown in Figure 7. Each transmission profile shows qualitatively the properties of the system, such as stability, separation behavior (floating, settling) and inter-particle interactions (e.g., coalescence, flocculation, etc.), so that these profiles can also be called fingerprint profiles of the dispersed system. The horizontal coordinates correspond to the position of the sample tube, with the top of the tube on the left and the bottom of the tube on the right; the vertical coordinates are the values of the transmittance. The spectral lines are shown from the initial spectrum (red) to the end spectrum (green) over time [17]. As shown in Figure 7, the first contour lines of the five emulsions were essentially straight at the beginning of the experiment, indicating that the droplets were uniformly distributed at the beginning of the emulsion.

The change of the transmittance of C under centrifugal force with time during the test is shown in Figure 7A. The ending spectrum remains smooth, indicating that C has good centrifugal stability. As shown in Figure 7C,E, the emulsions of VM and VT were gradually destabilized by the centrifugal force and the oil phase started to move towards the top of the sample tube due to its low density while the water phase remained at the bottom of the sample tube and the transmittance at the bottom gradually increased, suggesting that a frosted shape appeared at the top of the sample tube [17]. The change in transmittance of C/VM and C/VT under the centrifugal force is shown in Figure 7B,D. There is an improvement in the stability compared to VM and VT because the closer the sample is to the bottom of the tube, the thinner the transmittance region is and the more stable the emulsion will be [34]. It may be attributed to the fact that the presence of partial flocs reduces the density contrast between these droplets and the surrounding fluid, preventing the movement of individual droplets [26].

For a more intuitive comparison of changes in transmittance, the instability index was used for assessment. The instability index was between 0 and 1 and was used to quantify the process of emulsification, with 0 indicating no change in droplet concentration (very stable) and 1 indicating complete separation of dispersion (very unstable) [17]. As shown in Figure 7F, the instability index of C was 0.220, that of CM and VT were 0.386 and 0.371, and that of C/VM and C/VT were 0.251 and 0.226, respectively, and the experiments showed that the instability indices of the nano-emulsions formed by the composite essential oils, C/VM and C/VT, were reduced in comparison to those of CM and VT, which may be attributed to the fact that CM and VT have a larger droplet size. The larger droplet size of CM and VT may be due to the fact that the behavior of coalescence and agglomeration is more likely to occur under the action of centrifugal force.

### 3.7. Antioxidant Properties

**Figure 8:**
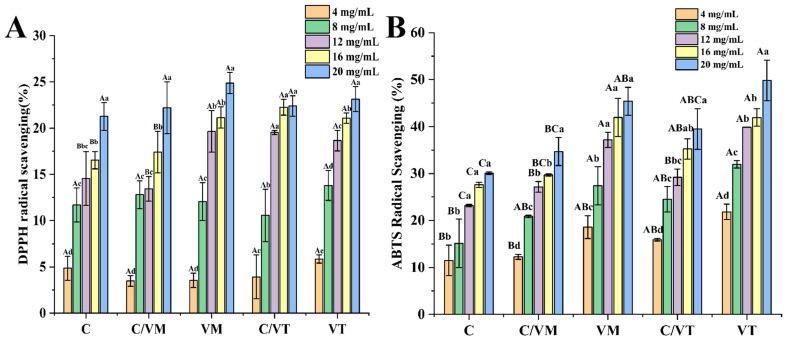
DPPH radical scavenging (**A**) and ABTS radical scavenging (**B**) of nano-emulsions. Lowercase letters indicate significant differences (*p* < 0.05) for the same nano-emulsion and capital letters indicate significant differences (*p* < 0.05) for different groups of nano-emulsions.

Droplets of nano-emulsions can increase the ability of lipophilic compounds to be biologically active by increasing the surface area per unit of mass [23]. The antioxidant properties were judged by the presence of polyphenolic substances in the essential oil, which reduced the number of DPPH free radicals [35]. As shown in Figure 8A, the scavenging ability of free radicals against DPPH was enhanced with the increase in mass concentration, and the scavenging energy rates of DPPH free radicals were 22.20% and 24.87% (*p* > 0.05) for C/VM and VM, and 22.38% and 23.13% (*p* > 0.05) for C/VT and VT, respectively, at the nano-emulsion mass concentration of 20 mg/mL. This indicates that the presence of flocs does not affect the clear ability of the nano-emulsion to DPPH radicals. In addition, the lower DPPH radical scavenging rate in this experiment may be due to the slow release of essential oils from the nano-emulsion, and, on the other hand, the interaction between antioxidants and stressors, the solubility of the samples in different systems and the steric selection of free radicals may lead to different antioxidant activities of the samples [36]. As shown in Figure 8B, the scavenging ability of nano-emulsions for the ABTS was positively correlated with the mass concentration of the emulsion. At the nano-emulsion mass concentration of 20 mg/mL, the ABTS radical scavenging energies of C/VM and C/VT were 34.67% and 39.46%, respectively, and those of C, VM and VT were 30.07%, 45.40% and 49.80%, respectively, which indicated that the vanilla essential oil had a better scavenging ability of ABTS radicals compared with that of the cinnamon essential oil (*p* < 0.05).

### 3.8. Stability of Complex Essential Oil Nano-Emulsions

Most of the emulsion formation process can be divided into primary homogenization and secondary homogenization in the secondary homogenization process (ultrasonic homogenizer, microfluidizer, high-pressure valve homogenizer, etc.), the main physicochemical processes occurring are the following: droplet breakage; droplet aggregation; emulsifier adsorption; droplet stabilization [37]. The water–oil interfacial tension of essential oils is one of the key factors affecting droplet size under the same homogenization and emulsifying molecular conditions [37]. For example, in the preliminary experiment, the water–oil interfacial tensions were measured for cinnamon essential oil and two types of vanilla essential oil (Madagascan and Tongan), and the values obtained were 20 mN/m, 24 mN/m and 29 mN/m, respectively. As illustrated in Figure 1, the results suggest that a lower water–oil interfacial tension is beneficial for emulsification and the formation of droplets of smaller droplet size.

Nano-emulsions are thermodynamically unstable systems and common physical stabilization mechanisms include gravitational separation, flocculation, coalescence, phase transitions and Ostwald ripening, and often these different physicochemical destabilization mechanisms tend to be interrelated [38]. During storage, one or more of these mechanisms dominate the composition and microstructure of emulsion droplets. Ostwald ripening is the growth of large droplets at the expense of small droplets and occurs more frequently in emulsions containing small droplets than in emulsions containing large droplets because the solubility of the dispersed phase increases as the droplet size decreases [39]. For example, in the present storage experiments, the significant growth of the cinnamon essential oil emulsion droplets can be attributed to Ostwald ripening, whereas Ostwald ripening was suppressed by the addition of the vanilla essential oil to the prepared composite emulsions. On the one hand, the smaller droplet size of C relative to the other emulsions means that Ostwald ripening proceeds faster in polydisperse emulsions; on the other hand, with the same conditions, it is the differences caused by different EOs. Ostwald ripening is usually negligible in emulsions with long-chain triacylglycerols as the oil phase, while in emulsions with short-chain triacylglycerols (e.g., flavor oils, essential oils) it tends to cause instability in the emulsions because of the high mutual solubility of their main components in water. The addition of vanilla essential oil reduces the solubility of the oil phase, which hampers the rate of Ostwald ripening [29,30]. However, emulsions with large droplet sizes can also suffer from other problems, such as flocculation, coalescence and gravity separation.

In the thermal stability experiments, the droplets in the emulsions were in constant motion due to the thermal energy, which increased the collision frequency between the droplets [26]. After heating at 85 °C and 100 °C for 20 min, the collision frequency between the droplets of the vanilla essential oil emulsions (VT, VM) increased and the droplet size increased significantly, probably due to the action of thermal energy, large droplets collided with each other and the aggregation and agglomeration of large droplets occurred. This was confirmed by the change in the droplet size of VM and VT observed in this study. The occurrence of agglomeration caused the fusion of the membranes of adjacent droplets, resulting in a decrease in the absolute value of the potential, whereas the increase in droplet size of the composite essential oil emulsions (C/VM, C/VT) after heating was still within an acceptable range.

The stability of composite essential oil nano-emulsions can be improved, to a certain extent, probably due to the complementary effect of the mixture of the two essential oils. Therefore, composite essential oil nano-emulsions may be a new approach to expand the application range of vanilla essential oil emulsions.

## 4. Conclusions

Vanilla (Madagascar, Tonga) essential oil and composite essential oil (vanilla/cinnamon 1:1 *v*/*v*) nano-emulsions were prepared using a microfluidizer processor using sodium caseinate and Tween 80 as the aqueous phases. Using droplet size, PDI and zeta potential as the indices of investigation, the prepared composite essential oil nano-emulsions C/VM and C/VT had droplet sizes of 99.07 nm and 98.89 nm, and zeta potentials of −34 mV and −30.53 mV, respectively. The TEM images showed that the nano-emulsion droplets were spherical in shape, and the presence of some flocs was observed in C/VM and C/VT. In the thermal stability experiments, after heating at 85 °C and 100 °C for 20 min, respectively, VM and VT showed sharp increases in droplet size (184.53 nm and 192 nm), while C/VM and C/VT still maintained good stability; after 24 days of storage at room temperature and 25 °C, C showed a phase separation phenomenon, and C/VM and C/VT did not show any obvious increases in droplet size. Combined with the LUM analysis, it can be seen that the centrifugal stability of C/VM and C/VT was better than that of VM and VT, probably due to the addition of cinnamon essential oil, and there was no significant effect on the scavenging rate of DPPH and ABTS radicals. Therefore, in future applications of vanilla emulsions, composite essential oils can be used to prepare emulsions to improve the centrifugal stability and thermal stability capabilities of vanilla emulsions and to increase their range of applications. Additionally, future work should further investigate the mechanism of flocculant formation in composite emulsions.

## Figures and Tables

**Table 1:** Turbidity values of nano-emulsions; lowercase letters indicate significant differences between different groups of nano-emulsions.

Samples	C	C/VM	VM	C/VT	VT
Turbidity (cm^−1^)	9.31 ± 0.05 ^e^	11.73 ± 0.47 ^c^	21.31 ± 0.02 ^b^	11.05 ± 0.12 ^d^	23.22 ± 0.35 ^a^

## Data Availability

The original contributions presented in the study are included in the article, further inquiries can be directed to the corresponding authors.
